# A Proteomic Survey of the Cystic Fibrosis Transmembrane Conductance Regulator Surfaceome

**DOI:** 10.3390/ijms241411457

**Published:** 2023-07-14

**Authors:** Melissa Iazzi, Sara Sadeghi, Gagan D. Gupta

**Affiliations:** Department of Chemistry and Biology, Toronto Metropolitan University, Toronto, ON M5B 2K3, Canada

**Keywords:** CFTR interactions, CFTR modulators, cystic fibrosis, interactome, surfaceome, peripheral quality control, PDZ domain, clathrin-mediated endocytosis, BioID

## Abstract

The aim of this review article is to collate recent contributions of proteomic studies to cystic fibrosis transmembrane conductance regulator (CFTR) biology. We summarize advances from these studies and create an accessible resource for future CFTR proteomic efforts. We focus our attention on the CFTR interaction network at the cell surface, thus generating a CFTR ‘surfaceome’. We review the main findings about CFTR interactions and highlight several functional categories amongst these that could lead to the discovery of potential biomarkers and drug targets for CF.

## 1. Introduction

The cystic fibrosis transmembrane conductance regulator (CFTR) protein is a cAMP-regulated anion channel that is not that widely expressed in human tissues and shows a tissue-specific expression pattern [1]. CFTR regulates numerous physiological pathways, such as the transport of Cl^−^ and HCO_3_^−^, glutathione and thiocyanate, immune cells, and the metabolism of lipids [2]. CFTR gene mutations are the cause for the fatal disease known as cystic fibrosis (CF) and CFTR-related disorders (e.g., recurrent idiopathic pancreatitis, congenital absence of the vas deferens) [3,4,5,6]. In the airways, CF is characterized by dehydration of the airway surface liquid, mucus plugging, and a vicious cycle of recurrent bacterial infections and inflammation [7]. CF lung inflammation is associated with an excessive neutrophilic response and lung tissue destruction [8]. To date, over 2000 CF variants have been reported, with ~90% of the CF population presenting with at least one copy of the most common mutation, a deletion of the phenylalanine in position 508 (∆F508) [9,10]. ∆F508-CFTR is characterized by its misfolding, retention in the endoplasmic reticulum and propensity for degradation [11,12]. Small-molecule correctors (i.e., VX-809 and VX-661) are partially effective in the rescue of defective processing and trafficking defects such as those exhibited in ∆F508-CFTR [13,14,15]. Ivacaftor (VX-770), a small molecule potentiator, increases channel open probability and restores channel activity at the cell surface [11,12,13,14], and has been approved for use in patients with gating defects (e.g., G551D-CFTR, and 97 other mutations) [16,17].

In 2019, Trikafta (VX445-VX661-VX770) was approved for use in over 90% of people with CF and is recognized as the most advanced approved therapy to treat patients [18,19]. Despite this advancement, current treatments are accompanied by comorbidities, and issues persist for the remaining patients with rare mutations that are currently not approved for Trikafta use [16,19]. This highlights the need to further our theratyping efforts and discover new drug co-targets for rare patient mutations. Furthermore, there are currently no treatment options for patients with nonsense mutations (e.g., W1282X and G542X) that result in large deletions of the CFTR gene [20,21].

CFTR function relies on the network of interacting proteins involved at every step of its synthesis and trafficking [11,22,23,24]. Several recent studies have utilized different proteomic techniques to generate broad datasets of the CFTR interaction network (interactome), and some have also investigated changes in the interactome due to mutations and after exposure to front-line therapies [9,11,12,23,25,26,27,28,29]. The aim of this review is to highlight connections between the different studies and consolidate the datasets with a focus on cell–surface interactions of CFTR. Despite recent advances in CF treatment, our knowledge of how CFTR is organized at the cell surface remains incomplete [9,23]. It is predicted that over half of the variation observed in CF lung function is likely to due to CFTR modifier genes (genes identified as associated with CF variability, severity, and/or treatment efficacy) [30,31,32,33]. However, these genes have been identified primarily via genome-wide association studies and transcriptomics [30,31,32,33], and may function at a different level of the CFTR interaction network. Furthermore, CFTR has understudied molecular functions in addition to Cl^−^ transport in different cell types, and the impact of this missing knowledge on the success of current CF therapies is unresolved [2]. This review hopes to support future functional CFTR studies, as parallel efforts to discover personalized therapies for rare patient mutations continue [9,34].

## 2. CFTR Interactomic Studies

**Figure 1:**
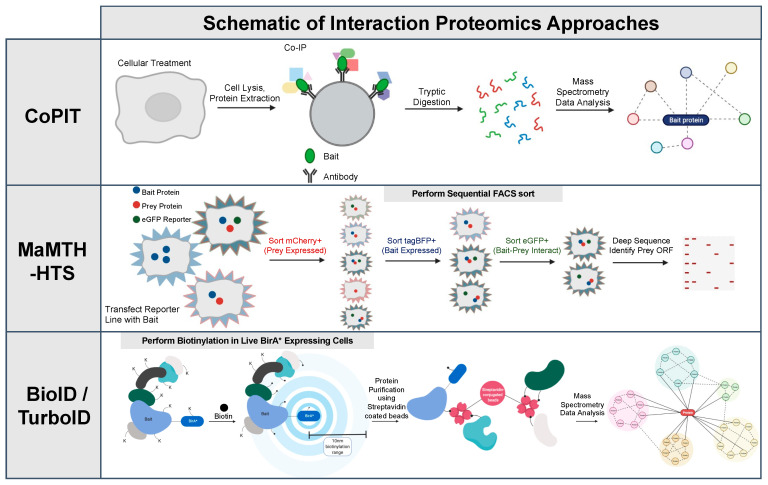
Schematic of different proteomic approaches used to investigate the CFTR interactome. Schematic for the workflow of the different proteomic approaches discussed in this review: Co-PIT, MaMTH-HTS, and proximity-dependent biotinylation methods (BioID and TurboID). Co-purifying Protein Identification Technology (Co-PIT).

**Figure 2:**
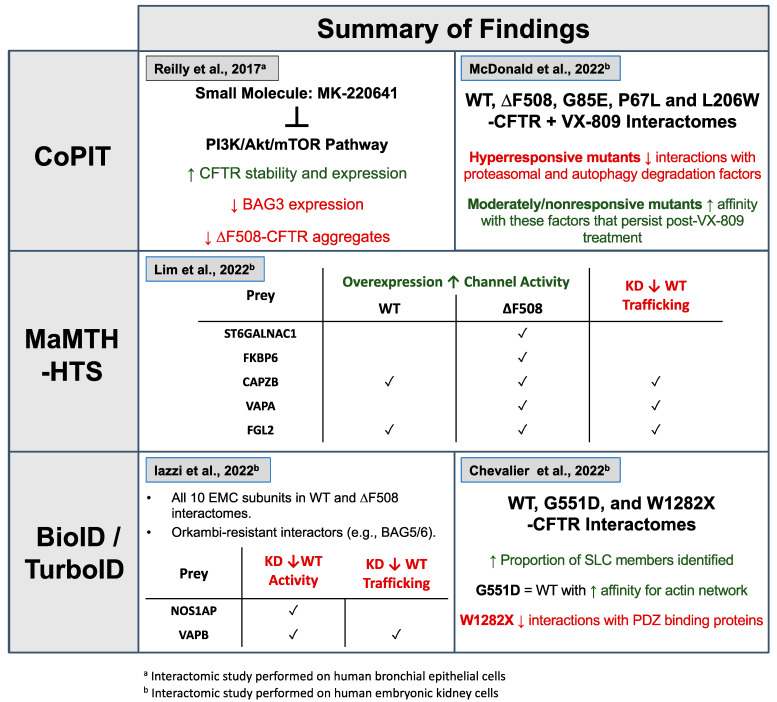
Summary of findings of different proteomic approaches used to investigate the CFTR interactome. Key details and findings for each of the five proteomic studies profiled in this review [11,25,27,28,29]. Arrows indicate an increase (↑) or decrease (↓) in WT or ∆F508-CFTR activity and/or trafficking. Check mark indicates the confirmed finding of each candidate interactor in the different studies. Created using BioRender.com.

There are currently three main proteomic techniques that have been utilized to study CFTR interactions (Figure 1 and Figure 2): (1) Co-purifying Protein Identification Technology (2) Mammalian Membrane Two-Hybrid; and (3) Proximity-Dependent Biotinylation.

The Co-Purifying Protein Identification Technology (CoPIT) is an immuno-precipitation (IP)-based proteomic-profiling approach of protein–protein interactions (PPIs) across different sample conditions [12]. It is a modification of a common method to study protein-complex-immunoprecipitation (IP), followed by mass spectrometry (MS) [35,36]. The Co-PIT methodology utilizes a solid support (i.e., sepharose beads) coupled to a highly specific antibody directed against a “bait” protein, in this case being CFTR [12]. The bait protein is used to precipitate protein complexes, and any captured proteins are subsequently digested into peptides before MS analysis [35]. Several factors can influence detectability in CoPIT/IP-MS methods, including the efficiency of solubilization of membrane-inserted proteins or the degree of stability of the interaction in post-cell-lysis steps [37,38].

CoPIT was used to study changes between the wild-type (WT) and ∆F508-CFTR interactomes and the effect of temperature-rescue (26–30 °C) in immortalized bronchial epithelial cell lines expressing WT-CFTR and ∆F508-CFTR [12,29]. WT and ∆F508-CFTR datasets constituted 504 and 506 interactors with 322 and 324 unique to WT and ∆F508-CFTR, respectively. This study noted CFTR associations to members of the mTORC2 complex, MAPKAP1 and RICTOR, amongst the ∆F508-specific interactors. These associations became attenuated in temperature-shift conditions, suggesting that inhibiting the PI3K/Akt/mTOR pathway may be important for the correction of ∆F508-CFTR [29]. Additionally, ∆F508-CFTR exhibited an association with Bcl2-associated athanogene (BAG) proteins 1–3. BAG3 was explored further due to the role it plays as a co-chaperone of Hsp70/Hsc70 for targeting misfolded/aggregated proteins for autophagic degradation. Inhibition of the PI3K/Akt/mTOR pathway with small-molecule inhibitor, MK-220641, resulted in an increase in CFTR stability, decrease in BAG3 levels, and decrease in ∆F508-CFTR aggregates (Figure 2) [29].

More recently, CoPIT was used in human embryonic kidney (HEK293) cells coupled with isobaric tandem mass tags to identify interaction network differences in CFTR mutants that are non-responsive (G85E), moderately responsive (∆F508), and hyper-responsive (P67L and L206W) to VX-809 treatment [28]. As in the Pankow study, ∆F508 was found to interact substantially more with components of the proteostatic and endoplasmic reticulum (ER) quality control machinery compared to WT-CFTR [28]. Moreover, the hyper-responsive P67L mutant had markedly lower associations with such components after VX-809 correction. This pathway restoration following VX-809 correction was replicated in L206W, another hyper-responsive mutant, and indicates that proteasomal degradation and autophagy interactions are associated with mutant responsiveness. Correspondingly, in a non-responsive mutant, G85E, treatment with VX-809 did not alter the mutant interactome in any quantifiable manner. This study suggested that VX-809 corrector binding may produce an inflection point in early biogenesis before the checkpoint to route for proteasomal degradation [28]. However, it is not apparent from an interaction(s) perspective why this does not occur in non-responsive mutants like G85E, and whether VX-809 fails to bind the G85E mutant.

### 2.1. Mammalian Membrane Two-Hybrid (MaMTH-HTS)

The Mammalian Membrane Two-Hybrid (MaMTH) involves the use of inactive fragments of ubiquitin, termed Cub and Nub, as sensors of PPIs [39,40]. These fragments remain inactive unless fused to two interacting proteins, whose proximity upon association drives reconstitution of the Cub and Nub into an active pseudo-ubiquitin molecule [39]. This newly reconstituted ubiquitin is then targeted by human-ubiquitin-specific proteases (DUBs) that cleave at the C-terminus of the Cub, releasing an artificial transcription factor (TF), which can then enter the nucleus and activate reporter gene transcription [39]. In an effort to map out the WT and ∆F508-CFTR, Lim et al. modified this previously described technique with a high-throughput screening (HTS) variant, now called MaMTH-HTS [27]. To generate the CFTR interactome, “bait” (CFTR and its variants) constructs were prepared by cloning the full-length proteins as fusions to a C-terminal bait tag consisting of the C-terminus of ubiquitin (Cub) and a GAL4 transcription factor linked by a P2A sequence to tag BFP [27]. The “preys” were 12,000 human open reading frames [41] cloned at their N-terminus to the N-terminal fragment of ubiquitin (Nub) and at their C-terminus to a P2A-mCherry tag. These were introduced via CRISPR into host HEK293 cells expressing GFP under the control of a GAL4-UAS reporter system. All constructs allowed for the titratable expression of heterologous proteins [27]. Host cells were systematically transfected with bait constructs, and interacting bait/prey pairs were detected via induced GFP expression. These were subsequently sorted by flow cytometry and identified by deep sequencing [27].

MaMTH-HTS revealed 224 and 269 candidate CFTR interactors for WT and ∆F508-CFTR interactomes, respectively. Functional characterization was performed for several PPIs (ZNF22, ST6GALNAC1, FKBP6, CAPZB, VAPA, XAGE3, FGL2) to assess the impact on CFTR trafficking and channel activity. FGL2 was the most important functional interactor uncovered from this and was orthogonally validated using co-IP. FGL2 is a secreted protein that is overexpressed in CFTR-knockout mice and may play a role in lung fibrosis [42]. Overexpression of FGL2 enhanced the functional output of both WT and ∆F508-CFTR channels. The siRNA knockdown of this gene resulted in a decreased trafficking of solely WT-CFTR. The results of this study indicate that the CFTR-FGL2 interaction could be important for CFTR function (Figure 2) [27].

### 2.2. Proximity-Dependent Biotinylation (PDB)

Proximity-dependent biotinylation (PDB) coupled to MS has been developed to label proximal proteins with biotin [35,36,43]. Proximity-dependent biotin identification (BioID), APEX, and their derivatives have been widely used to define a protein of interest’s (bait) interactome [35,36]. Both BioID and APEX are based on the genetic fusion of a PDB enzyme to a bait and this induces the covalent tagging of proteins in the bait’s vicinity [35,36]. Tagged proteins are subsequently captured after cell lysis and identified by MS, negating the need to maintain intact structures during lysis and purification [35,36]. The BioID technique fuses a mutant form of an E. coli biotin conjugating enzyme, BirA R118G (BirA*), to a protein of interest (bait). BirA* allows for the permanent tagging of the lysine residue on proximal proteins within a ~10 nm radius of the bait [36,43,44]. BioID is distinguishable from techniques like MaMTH because it detects endogenously expressed preys and is also distinguishable from co-IP techniques because it reports interactions occurring in living cells. Two variants of BioID, miniTurbo and TurboID, have been developed to have higher catalytic affinity and allow for biotin labelling to occur over the span of minutes instead of several hours [35,45]. APEX can covalently biotinylate proximal proteins on tyrosine residues by using modified ascorbate peroxidases to oxidize biotin-phenol to produce highly reactive phenoxyl radicals [35]. Both BioID and APEX can undergo harsh lysis conditions and generate an interactome in the context of a living cell; however, APEX requires hydrogen peroxide treatment, which can have important unwanted cellular effects [35].

BioID was recently used to characterize the interactomes of WT and ∆F508-CFTR in response to treatment with Orkambi (3 µM VX-809 + 1 µM VX-770) and proteasome inhibitor, MG132 [11]. MG132 was used to reduce the premature degradation of ∆F508-CFTR by inhibiting the proteasome [11,46]. The group used the standard form of BioID, which allows for the labelling of proximity interactors over the span of 16 h, approximately the length of a cell cycle [36]. The study focused on HEK293 cells stably expressing a CFTR construct with an N-terminus BirA* tag [11]. The WT and ∆F508-CFTR constituted 474 and 626 interactors, respectively [11]. Similar to the trend seen with Co-PIT studies [12,26], and the MaMTH study [27], off-pathway ∆F508 interactions consisted primarily of protein folding and degradation components, and these were attenuated upon VX-770 + VX-809 addition [11]. Two candidate membrane traffic interactors, VAPB and NOS1AP, were identified, and their impact on WT-CFTR trafficking and functional activity was characterized [11]. Notably, the BioID approach revealed that WT CFTR was extensively associated with vesicle trafficking machinery. While some of these were also identified with other proteomic approaches (Supplementary Dataset S7), we suggest that differences in the interactomes may relate to the detectability or preservation of transient interactions during membrane trafficking steps.

Using BirA* tags at opposite ends of a protein can sometimes provide additional interaction information about the bait protein, depending on the accessibility of its various interactors to the biotinylating enzyme [47]. We compared unpublished N-terminally (N-CFTR) and C-terminally (C-CFTR) tagged FLAG-BirA* datasets that were generated in HEK293 Flp-In T-REx cells as part of an earlier study (Supplementary Figure S2A) [11]. HEK293 cells have been utilized extensively for CFTR interactomics since they express little to no endogenous CFTR [11,25,27,28]. N-CFTR and C-CFTR generated 297 and 70 candidate interactors, respectively (Supplementary Dataset S1 and S2), and those corresponding to the plasma membrane (PM) Gene Ontology (GO) category made up nearly half of the combined interactomes (~43%) (Supplementary Figure S2B). Fourteen previously validated interactors were also identified (BioGRID database). [48] The N- and C- interactomes shared 50 common interactors (Supplementary Figure S2). The C-terminal fusion may impede binding sites for several well-characterized CFTR interactors that affect its trafficking and organization at the cell surface [23,49]. Consistent with this, several known PDZ-containing proteins are present in the N-terminally tagged CFTR BioID dataset, while these interactions are absent in the C-terminally tagged dataset (Supplementary Figure S2C).

More recently, another proximity biotin labeling study utilized TurboID and APEX2 on WT-CFTR and compared the interactome to a structurally unrelated potassium channel, KCNK3, and to two known mutants, G551D and W1282X, in HEK293 cells [25]. The major difference between the different techniques is the labelling time between traditional BioID (12–24 h) to TurboID and APEX2 (minutes) [36,50]. The interactomes generated 1002 and 965 high-confidence interactors using the APEX2 or TurboID approach, respectively, and exhibited a prey overlap of approximately 50% [25]. The use of proximity labelling identified a greater proportion of SLC transporters than reported in previous co-IP-based methods, and this is likely due to the permanent labelling of proximal proteins at the PM that otherwise would not survive harsh lysis conditions. APEX2 and TurboID were also used to determine differences in enriched proteins in the interactomes for CFTR mutants, G551D (22 and 9) and W1282X (101 and 280), when compared to WT-CFTR [25]. Notably, very few differences were observed between WT and G551D interactomes. However, the latter did exhibit a higher affinity for the actin network, which is consistent with previous reports [51]. The W1282X-CFTR interactome showed more differences when compared to WT-CFTR, including the expected loss of PDZ binding proteins due to the truncation of the NBD2 and the C-terminal domains (Figure 2).

## 3. Mapping the CFTR Surfaceome

**Figure 3:**
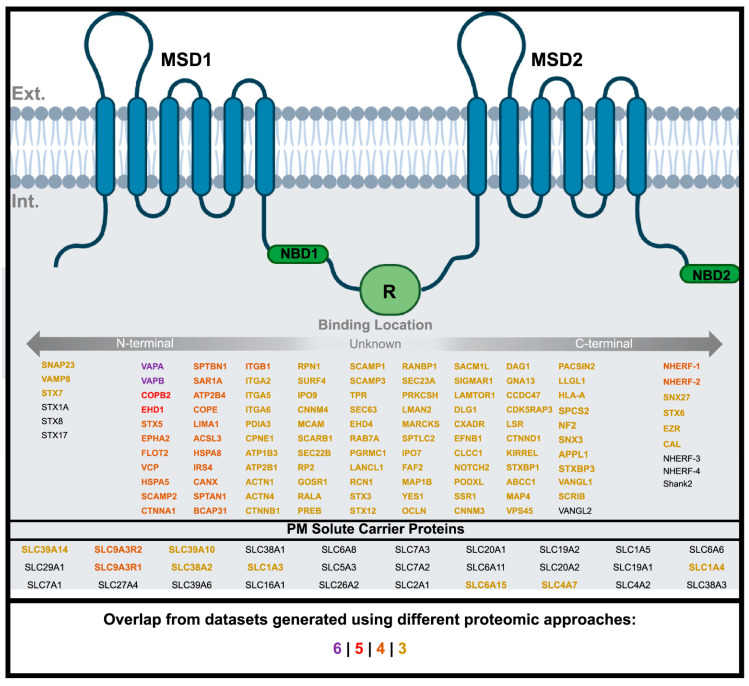
WT-CFTR Surfaceome. Depiction of CFTR protein structure consisting of two nucleotide binding domains (NBD1/2), membrane spanning domains (MSD21/2), and regulatory domain (R) inserted in the PM. Interactors generated from the different proteomic approaches that are associated with the PM according to Gene Ontology (Supplementary Dataset S5), arranged according to their predicted (if known) site of binding to CFTR. Degree of overlap of interactors between different approaches is represented by colour: purple (6), red (5), orange (4), and yellow (3). Created using BioRender.com.

The surfaceome is a broad term that represents the interactome of cell-surface proteins. Proteomic approaches aim to characterize the network of interacting proteins in a cell in the context of a protein of interest. The WT-CFTR surfaceome can create a specific signature of interacting proteins and represents a foundational dataset for hypothesis generation and comparative testing [34,52]. If we consider all interacting proteins that were identified in the proteomic studies outlined here, we obtain 924 total overlapping interactors for WT-CFTR (present in at least 2 of the reviewed proteomic studies) (Supplementary Dataset S3) [11,12,25,27,28,29] and 10 (all Co-PIT; Supplementary Figure S1A) and 105 (all proximity-based) overlapping interactors (Supplementary Figure S1B). Of the 924 interactors, 315 correspond to the PM GO category [53]. We define the latter as the “CFTR Surfaceome” (Supplementary Dataset S4). A higher confidence dataset that comprises preys found in at least three of the reported interactomes totals 113 interactors (Figure 3; Supplementary Dataset S5) [11,12,25,27,28,29].

### 3.1. CFTR Surface Organization and PDZ Domain Effectors

**Figure 4:**
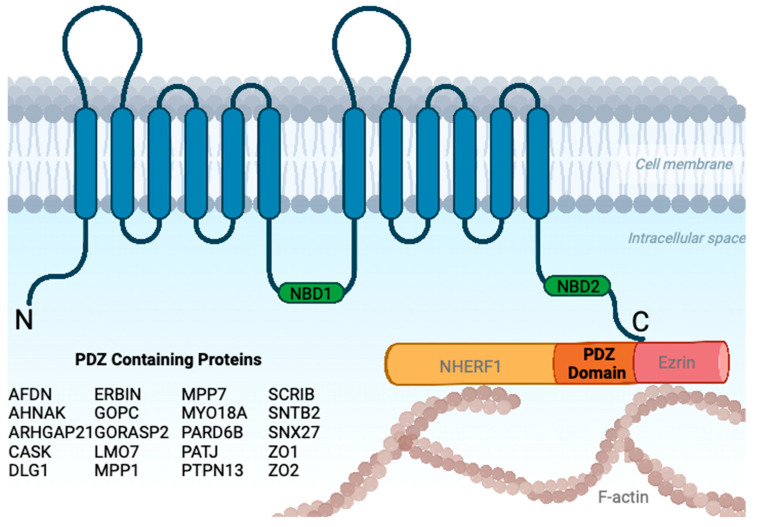
WT-CFTR surfaceome includes set of PDZ-domain-containing proteins. Candidate interactors that contain PDZ-domains. Created using BioRender.com.

PDZ (PSD95, Dlg1, ZO-1 binding motif) domains act as protein-interaction domains that are specialized for binding short peptide motifs located at the extreme carboxy (C) termini of proteins [54]. There are typically multiple PDZ domains located on a single protein, enabling it to behave as a scaffold for other PDZ-domain containing proteins [55]. The C-terminus of CFTR contains the PDZ interaction domain (Figure 4) [56] and many of these interactions are lost if the C-terminal end of CFTR is modified (see Supplementary Figure S2). CFTR is tethered to the actin cytoskeleton at the apical PM in epithelial cells through its interaction with NHERF1/2 through its PDZ domain [57] and the actin-binding protein ezrin [57,58]. Furthermore, the first 20 N-terminal amino acids of CFTR are known to interact with FLNA/B, an interaction known to stabilize CFTR at the PM by anchoring to the actin cytoskeleton [59,60,61,62]. FLNA and FLNB are both found in the consensus surfaceome (Supplementary Dataset S3).

Known C-terminal end CFTR interactors include proteins with PDZ domains—NHERF1, NHERF2, EZR, STX6, and CAL (GOPC)—and are present in the CFTR surfaceome (Figure 4) [23,63]. It must be noted that Shank2, NHERF3 (PDZK1), and NHERF4 (PDZK2) are also well characterized [23,49] in terms of their interactions with the C-terminal end of CFTR but are not detected in these studies. Golgi Reassembly Stacking Protein 2 (GORASP2 or GRASP55) is known to interact with core glycosylated CFTR via its PDZ domain and mediates unconventional CFTR trafficking [64,65]. The transgenic expression of GRASP in ΔF508-CFTR expressing mice has been shown to restore channel function with no observable toxicity [24]. The previously mentioned GOPC protein contains two coiled-coil domains and one PDZ domain that are known to interact with the C-terminus of CFTR [66,67]. The interaction between GOPC:CFTR reduces surface CFTR through its endocytic recycling by targeting it for lysosomal degradation, and inhibiting this interaction helps stabilize CFTR at the PM [67]. Inhibiting GOPC was also reported to have an additive increase in the surface quantity of CFTR in the presence of front-line small molecular correctors, VX445/VX-809 [67]. The Scribble cell polarity module, comprising Scribbled (Scrib), Discs-large (Dlg), and Lethal-2-giant larvae (Lgl), has a tumor suppressive role in mammalian epithelial cancers [68]. SCRIB, DLG1, and Afadin are novel PDZ effectors for CFTR, as predicted by the surfaceome. The scribble module protein is involved in cellular processes including cell adhesion, membrane trafficking, cell migration, and cellular signalling [68]. Furthermore, Afadin (AFDN) is a third novel PDZ effector for CFTR (Figure 4). AFDN plays a role in the establishment and maintenance of cell–cell contact sites and is a unique RAS GTPase effector [69]. The latter may be important for the correctional rescue of ∆F508-CFTR [29]. A number of studies using image correlation spectroscopy and single-molecule tracking, combined with molecular or cytoskeletal perturbations, have yielded key insights on the nanoscale organization of CFTR at the cell surface [70,71,72,73,74]. The current view is that CFTR is localized in at least two populations, one that is in nanoscale clusters and another that is diffusely distributed [75]. Clustered CFTR channels have relatively slow, confined movements over a small spatial scale, consistent with their localization in lipid microdomains, whereas the diffusely distributed population has transport dynamics on larger spatial scales that reflect CFTR movements both inside and outside microdomains [76]. Importantly, clustering occurs not because of the aforementioned CFTR interactions with PDZ domain proteins or actin and filamin A, but is primarily lipid-interaction-driven. It remains to be seen how the CFTR surfaceome could be involved in or affected by this dynamic nanoscale organization, and this interplay of lipids and CFTR (and presumably its interactors) promises to be an exciting area of future study. In the context of ceramide-rich domains [76], SMPD4, a neutral sphingomyelinase, that generates ceramide from sphingomyelin, was detected in two CFTR interactomes (Supplementary Dataset S3).

### 3.2. The CFTR Surfaceome Includes a Diverse Set of Membrane Transporter Interactions

**Figure 5:**
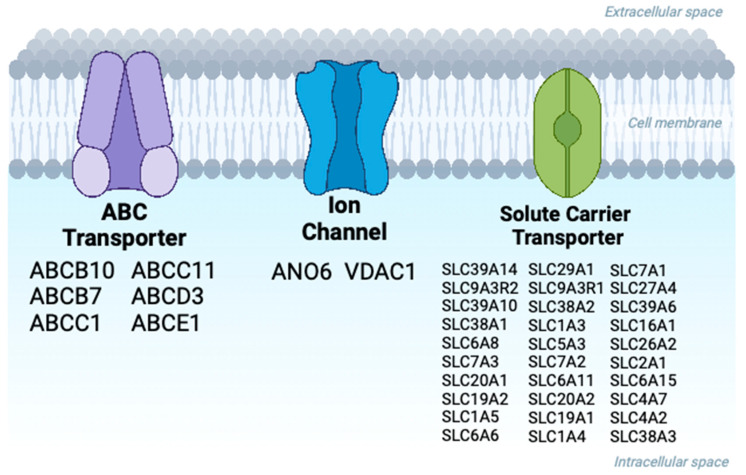
WT-CFTR surfaceome includes a diverse set of membrane transport interactors. Candidate interactors derived from different proteomic approaches that correspond to ion channel proteins or previously reported solute carrier transporter proteins [11,25]. Created using BioRender.com.

ABC transporters are multi-domain membrane-spanning proteins responsible for the transport of substrates across the membrane, regulated by ATP hydrolysis, and essential for homeostasis [77,78]. CFTR (ABCC7) is one of the most recent evolutionary members of the ABC superfamily and is unique in its functioning as a chloride channel [79]. The CFTR channel has been shown to functionally interact with and regulate the epithelial sodium channel (ENaC) [80,81], the outwardly rectifying Cl^−^ channel, and the renal outer medullary potassium channel, thereby contributing to cellular ion homeostasis [82]. Also, CFTR HCO_3_^−^ secretion in the airways, pancreas, salivary gland, intestine, and reproductive organs is also associated with the activity of the anion exchanger 2 (SLC4A2) and electrogenic Na^+^/HCO_3_^−^ cotransporter (SLC4A4) [2]. However, the exact mechanism of CFTR interdependency of these channels is still not fully understood, and various hypotheses including the direct or indirect interaction of channels, or co-regulation via adapter proteins have been proposed [83]. Unfortunately, HEK293 cells do not express most of these channels at levels that are easily detectable in interactomes. The Cl^−^/HCO_3_^−^ exchanger SLC4A2 is an exception to the latter trend, and is detectable in two interactomes, while another HCO_3_^−^ transporter, SLC4A7, is present in three interactomes (Figure 5). The dataset presented here should provide a starting point for further investigations into the role of these CFTR- membrane transporter interactions.

In general, PDB methods have identified a larger cohort of solute carrier (SLC) transporters [11,25]. Of the 54 SLC transporters reported, 30 were statistically enriched at the PM and 27 are present in the CFTR surfaceome (Supplementary Dataset S6). Some SLCs have been associated with phenotypic diversity in patients with CF [84] but are unfortunately not expressed well enough in HEK293 cells to be detectable. SLC9A3R1 (NHERF-1) and SLC9A3R2 (NHERF-2) are well-characterized interactors of CFTR and are present in four of the reported datasets [23,49]. Seven SLC interactors with uncharacterized roles in CFTR function were reported in three proteomic studies (SLC39A14, SLC39A10, SLC38A2, SLC1A3, SLC6A15, SLC4A7, SLC1A4) (Figure 5; Supplementary Dataset S6) [11]. SLC39A14 is a manganese influx transporter that is highly expressed in the liver and small intestines and its loss of function is associated with the childhood-onset of dystonia-parkinsonism [85]. SLC39A10 (ZIP10) is identified as a key zinc transporter in hematopoiesis and its inhibition has been linked to treating STAT-3-activated cancers [86,87]. SLC38A2 is a neutral amino acid transporter and its expression is needed in both osteoblast differentiation and bone formation in mice [88].

Two additional channel proteins were present in the CFTR surfaceome (Figure 5). Anoctamin 6 (ANO6 or TMEM16F) is a Ca^2+^-activated chloride channel and phospholipid scramblase [89]. The expression of ANO6—and its more characterized family member ANO1—induces increased membrane trafficking and exocytosis of volume-activated chloride channels, such as CFTR [90]. CFTR is known for its proapoptotic effects and growing evidence is linking ANO6 to be a regulator of cell death [89,90]. Similarly, VDAC1 (Voltage-dependent anion-selective channel 1) is associated with mitochondrial dynamics including regulating apoptosis and autophagy [91]. It has been speculated that VDAC1 could be regulated and closely related to CFTR function as CFTR inhibitors have been reported to decrease VDAC1 expression [91]. Lastly, six members of the ABC transporter superfamily were found in the surfaceome (ABCB10, ABCB7, ABCC1, ABCC11, ABCD3, ABCE1) (Figure 5). Multidrug resistance ABC transporter (ABCC1 or MRP1) has the ability to translocate substrates such as tobacco-specific carcinogens that can compete with cAMP translocation and in turn is speculated to modulate CFTR channel activation [92]. ABCC11 (or MRP8) was previously used as a clinical marker for CF and termed the “CF antigen” due to its elevated concentrations in CF patients’ serum [93].

### 3.3. The ∆F508-CFTR Interactome

**Figure 6:**
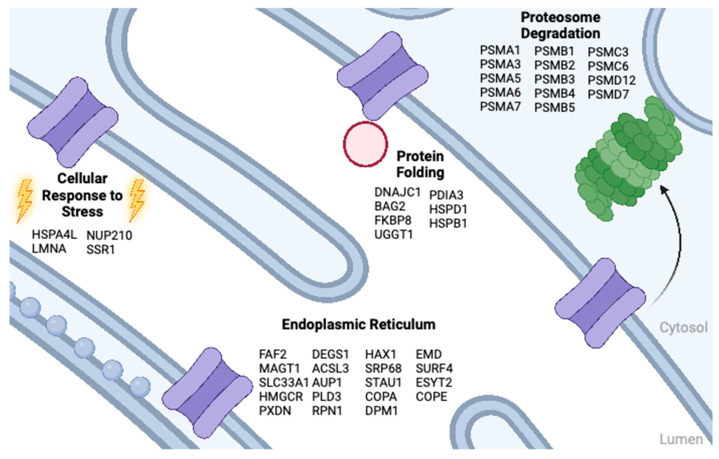
∆F508-CFTR interactome. Overlapping interactors derived from different ∆F508-CFTR interactomes that are associated with (1) cellular response to stress; (2) endoplasmic reticulum; (3) protein folding; and (4) proteosome degradation. Created using BioRender.com.

In most cell types, CFTR folding is inefficient [94]. Depending on the cell type, up to approximately 30% of wild-type protein and 99% of the most common inherited ΔF508 mutant are degraded via the endoplasmic-reticulum-associated degradation pathway (ERAD) [95,96,97]. During early synthesis steps, CFTR is recognized by the Hsc70 chaperone and this step commits CFTR to the PM or for lysosomal degradation [98]. When mining the proteomic studies outlined here for the ∆F508-CFTR interactome, we obtain a total of 90 consensus preys (Supplementary Dataset S3) [11,27,28,29]. Of these, 46 correspond to protein folding (7; GO:0006457), the cellular response to stress (4; GO:0033554), proteosome degradation (14; WP183), and the endoplasmic reticulum (21; GO:0005783) [53] (Figure 6). Interestingly, several studies note a subset of ∆F508-CFTR-specific interactors that persist upon VX-809 correction [11]. These include proteasome and co-chaperone subunits: PSMC1, PSMD11, PSMB8, BAG3, DNAJB2, SURF4, and ERH [12]. The mutant CFTR interaction with BAG proteins, in particular, appears to correlate with folding correction [26,28,29,99]. BAG2 associates with folding mutants of CFTR (∆F508, P67L, L206W) and VX-809 hyper-responsive mutants such as P67L are less likely than moderately responsive mutants (∆F508) to have this interaction persist post correction [28]. BAG5 and BAG6 exhibit a preferential binding to ∆F508-CFTR; however, these interactions persist post-VX-770 + VX-809 treatment [11].

J proteins and Hsp40 co-chaperones interact with CFTR during the initial translation stages [100], and members, such as DNAJB12, have been shown to triage ∆F508-CFTR through proteasomal degradation [101]. DNAJB12 associates preferentially with ∆F508 and P67L-CFTR, and this association is attenuated upon VX-809 treatment [28]. Similarly, DNAJA3 and DNAJA4 exhibit preferential binding to ∆F508-CFTR that becomes attenuated when treated with VX-770 + VX-809 [11]. An excellent review describes recent progress in discovering CFTR proteostasis regulators [102].

### 3.4. CFTR Surfaceome and Peripheral Quality Control

Partially unfolded CFTR at the PM (e.g., corrected ∆F508-CFTR) is subjected to ubiquitination in post-Golgi compartments and recognized by ubiquitin-dependent endosomal sorting machinery to reroute the channel from the recycling pathway toward lysosomal degradation [98,103]. While chaperone machinery (i.e., Hsc70/Hsp90) functions to maintain the properly folded CFTR conformation at the PM, structurally unstable CFTR (such as of misfolded and/or rescued mutant CFTR protein) has a reduced half-life [104,105]. This is due to modifications caused by members of the chaperone-dependent ubiquitination machinery in a process referred to as the peripheral quality control (PeriQC) system [104,105,106]. The PeriQC system removes non-native proteins from the PM for lysosomal degradation by ubiquitination to preserve the cell permeability barrier [106].

The chaperone-dependent ubiquitin ligase CHIP (carboxy terminus of HSP70-interacting protein) is the first E3 Ub ligase identified in the PeriQC mechanism of CFTR [105]. The prolonged association of corrected CFTR with the Hsc70/Hsp90 chaperone complex is thought to recruit CHIP and result in the ubiquitination of CFTR [105]. Two additional PeriQC members were recently identified as well: RFFL (CARP2) and RNF34 (CARP1) [104,105,106]. Amongst all overlapping WT-CFTR interactors, 56 were statistically enriched in the GO category for ubiquitin-like protein ligase binding (GO:0044389) (Supplementary Dataset S7). Previously characterized CFTR chaperones are present in this dataset (i.e., DNAJA1, BAG2). However, the only known PeriQC component identified in this dataset is CHIP.

Additionally, the role of deubiquitinating enzymes (DUBs) is emerging as an important process for the regulation of CFTR stability beyond the Golgi apparatus [105]. USP19 is a DUB acting on ∆F508-CFTR, allowing it to bypass ERAD [105,107,108]. Furthermore, USP10 has been reported to reverse the ubiquitination of WT-CFTR, enabling CFTR to bypass lysosomal degradation and return to the PM [105,107,109]. The role of DUBs and their potential to stabilize CFTR at the PM are not fully understood and the mechanism of PeriQC is still unclear. Interestingly, USP19 is found in the WT-CFTR surfaceome, and several other DUBs (USP5, USP7, USP9X, USP14, and USP19; Supplementary Dataset S7) are also present but have not been characterized.

### 3.5. CFTR Surfaceome and Vesicle Trafficking

A total of 87 of the 315 surfaceome members were statistically enriched in the GO category for vesicle-mediated transport (GO:0016192) (Supplementary Dataset S7). Some known vesicle trafficking interactors are present in at least three of the datasets: VAMP8, SNAP23, and STX7 [110,111]. It has been previously reported that there is a marked reduction in preys annotated in this category in the ∆F508 mutant [11], consistent with its failure to enter the CFTR-PM pathway. Interestingly, VAPA and VAPB were the only interactors to appear in all six of the interactome datasets examined (Figure 3). VAPs are generally ER and Golgi-localized membrane-anchored proteins that participate in vesicle trafficking and control ER-PM contact sites [112,113]. VAPs have also been proposed to regulate CFTR biogenesis and inhibit the degradation of mutant CFTR [112]. Two studies have identified this interaction in their proteomic datasets and further characterized its importance on CFTR trafficking and function [11,27]. Interestingly, VAPA and VAPB interactions with ∆F508-CFTR are attenuated compared to WT-CFTR but restored upon treatment with VX-770 + VX-809. Knockdown of VAPA/B resulted in a significant decrease in WT-CFTR trafficking and was important for CFTR channel activity [11,27].

Additionally, COPB2 (COPI Coat Complex Subunit Beta 2) and EHD1 (Eps15 homology domain 1) are PM-associated preys that appeared in five of the interactome datasets (Figure 3). COPB2 has been implicated to play a critical role in CFTR trafficking to the PM [114]. WT-CFTR has been shown to accumulate in endosomal recycling compartments marked by EHD1 and become redistributed onto the cell surface upon phosphorylation by protein kinase A (PKA) [115].

### 3.6. CFTR Surfaceome and Clathrin Endocytic Machinery

The surfaceome contains 30 proteins that correspond to the GO category for clathrin-dependent endocytosis (GO:0072583) (Supplementary Dataset S7). It has been long recognized that clathrin-dependent internalization of WT-CFTR is the main pathway for CFTR recycling at the PM [103]. The carboxy-terminal tail of CFTR contains conserved tyrosine-based (YXXϕ motif) signals that regulate its entry into clathrin-coated pits (CCPs) via the adaptor AP2 complex [103]. The internalization of mature CFTR through clathrin-mediated endocytosis (CME) acts as a quality control mechanism [98]. Considering the rapid internalization in some cell types (up to 10%/minute) and slow translational rate, a focus on re-targeting endocytosed CFTR back to the cell surface is of utmost importance [103,116,117].

**Figure 7:**
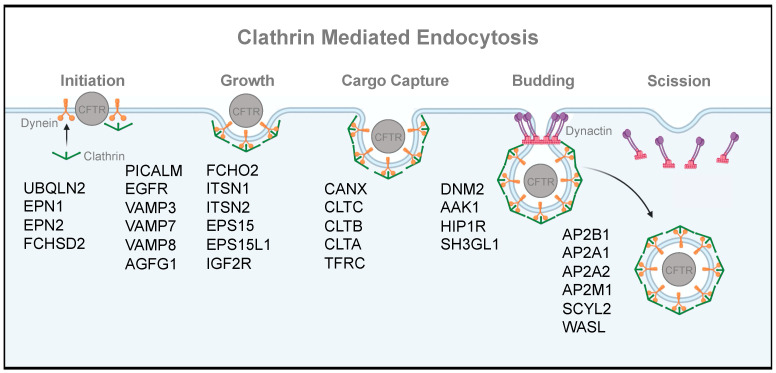
WT-CFTR surfaceome reveals subunits of the clathrin-mediated endocytic pathway. Depiction of steps involved in clathrin-mediated endocytosis from clathrin-coated pit assembly to clathrin-coated vesicle scission, overlaid with candidate interactors generated from the different proteomic approaches. Locations are approximated based on where these proteins are predicted to be involved in clathrin-coated vesicle formation. Created using BioRender.com.

Remarkably, the curated list of WT-CFTR interactors show an extensive association with different steps of CME, from CCP initiation to clathrin-coated vesicle (CCV) budding and scission. This is probably due to the high recycling rate and retention in endosomal routes [118]. Here, we briefly survey the list of interactors and their roles in CME, as relatively sparse information exists on their individual effect on CFTR function. The assembly of CCPs is an intricate process that begins with clathrin triskelions being recruited to the PM by the AP2 complex and PIP2 (PtdIns(4,5)P2) to initiate pit assembly (Figure 7) [119]. AP2 A1/A2/B2 subunits were readily detected in the surfaceome, and experimental evidence exists for CFTR interacting with the alpha AP2 subunit in intestinal cells [120]. FCH/F-BAR and Double SH3 Domain-Containing Protein (FCHSD2) is a member of the mild curvature-generating F-BAR family of proteins, whose prototypical members of FCH And Mu Domain Containing Endocytic Adaptor proteins (FCHO1/2) have been shown to function in the early stages of CCP initiation and stabilization [121,122]. Clathrin assembly lymphoid myeloid leukemia (CALM/PICALM) is also necessary for clathrin assembly at the PM by binding to AP2 and clathrin [123]. Epsin (EPS15) and intersectin (ITSN) are initiator proteins that form a complex with FCHO to recruit AP2 and other adaptor proteins that in turn recruit clathrin subunits (CLT A/B/C) [119]. Furthermore, Dynamin-2 (DYN2 or DNM2) is one of three isoforms and is the only one that is ubiquitously expressed in cells and functions as a fission apparatus at the necks of invaginated CCPs, and regulates early steps of CCP maturation [119,124]. Loss of DNM2 inhibits endocytosis and enhances the surface expression of CFTR [66]. Actin polymerization during the late steps of CME help release CCVs from the PM and into the cytoplasm [125]. HIP1R links the CME machinery to the actin skeleton [119], while WASL (WASP Like Actin Nucleation Promoting Factor) is essential for the formation of CCVs [125]. More peripheral CME regulators include SCYL2, which phosphorylates AP2 and target membrane receptors for lysosomal degradation [126,127]. Lastly, Ubiquilin (UBQLN) is known to increase the cell surface expression of receptors (e.g., G-protein-coupled receptors) and has been suggested to be a negative regulator of endocytosis [128]. 

It is important to understand how mutations in CFTR affect its endocytosis. To date, three CF patient mutations (N287Y, R31C, R31L) have been identified as having endocytic defects, and all are associated with a mild clinical phenotype. Interestingly, all three result in an enhanced CME of CFTR, leading to lower PM CFTR levels, but the precise mechanism is unclear [117]. While several other CF mutations have been associated with a lower steady-state CFTR level at the PM, endocytic rates of these mutants are rarely established, so it is difficult to distinguish between forward trafficking, recycling, and endocytic defects that result in this phenotype. Complicating this further, most non-polarized cellular models do not recapitulate the basal–apical transcytosis of CFTR in secretory epithelia [129]. The extensive association of the surfaceome with CME machinery suggests that this is a direction worthy of further, more careful investigation.

### 3.7. The CFTR Surfaceome and Innate Immunity

The CFTR protein is also expressed by immune cells, and the loss of functional CFTR in CF may result in dysregulation of their functions [2]. These include an impaired bacterial killing and degranulation response in neutrophils, defective phagocytosis in macrophages, a disruption in T- and B-lymphocyte response, and an intrinsic hyperinflammatory phenotype [130]. Though controversial, there is growing evidence that inflammation is a primary consequence of the CFTR defect, rather than purely a response to airway infection [82,130,131]. A survey of the CFTR surfaceome indicates a significant overlap with components of innate immunity, although a large portion of the overlap constitutes membrane trafficking regulators (Supplementary Dataset S7). Specifically, ANXA1 and ATP6AP2 stand out as direct regulators of the immune response and are also interesting because they were identified in a proteomic study as differentially expressed in CFTR −/− neutrophils from newborn piglets [131]. ANXA1 (Annexin A1) is a well-described effector of anti-inflammatory processes [132] and is downregulated in CF mice and CF human patients [131,133]. By contrast, ATP6AP2 is upregulated in CF piglets [131] and is frequently associated with markers of lung inflammation [134]. ATP6AP2 is a renin receptor that activates the renin–angiotensin system and intracellular signal transduction, thereby enhancing inflammation. It also functions in lysosomal acidification and affects neutrophil degranulation [134]. The presence of these innate immune modulators in the CFTR surfaceome suggests a more direct relationship and crosstalk that warrants further investigation.

## 4. Future Perspectives and Closing Remarks

The success of current small-molecule drugs in correcting CFTR folding, trafficking, and membrane localization is proof of the power of gene-function studies, and the value of elucidating protein interactions. Reliable interaction data can help distinguish between primary and secondary defects arising from CFTR mutations in the numerous cellular contexts where this protein functions, and lead to the discovery of important gene modifiers for CF. We note that four genes highlighted (SLC9A3, HLA, IGF2BP2, CDKAL1) in a recent review on CF modifiers [33] are present in the CFTR surfaceome presented here. Additionally, the development of next-generation compounds targeting molecular components of the CFTR interaction landscape could be a useful strategy to optimize combination therapies for those patients with mutations that are poorly responsive to current treatments. Notably, proteomic and functional genomic studies have uncovered proteostasis regulators such as DUBs, ubiquitin ligases, and folding chaperones that may hold significant promise as targets of ‘∆F508 amplifier’ drugs [102]. Another class of CFTR interactors, the SLC transporters, plays a crucial role in controlling the transport and net flux of drug absorption into cells [135]. The idea of targeting SLC transporters in combination therapy modalities has been gaining traction and there are drugs already available [136]. Yet, other classes of CFTR interactors such as those corresponding to membrane trafficking and innate immunity await further characterization and mechanistic investigation.

The approaches used in CFTR interactomic studies that have been profiled here have their own distinct advantages (e.g., detection of endogenous interactions, detection in a living cell, scalability) and limitations (e.g., size of probes, overexpression of bait protein, lack of a catalytic step) [11,25,27,28,29,36]. Unsurprisingly, there is a low degree of overlap when comparing data between individual studies (Supplementary Figure S1A,B) [11,27,137]. Variances almost certainly arise from the different interaction sensors employed and the use of different cell types. Therefore, the interactomes generated using different proteomic approaches are best viewed as complementary data, which, in some specific cases, can provide meaningful overlap, and, in many cases, have the power to generate insight into CFTR protein interactions in their own contexts. Interactomic studies, regardless of the approach used, are shifting toward using these data as intra-comparative tools to generate broad molecular fingerprints of the drug response in CFTR mutants. This facet of CFTR investigations, which has become increasingly accessible in modern laboratories, will accelerate the discovery of more personalized treatment options in the CF population. Furthermore, in the near term, applying interaction biosensors and interactome technology to discover key interactions in diverse physiological settings where CFTR is expressed, as well as in stem-cell-derived tissue models of CF, is certainly a worthwhile and achievable milestone.

## Data Availability

The data presented in this study are available in the [Supplementary Material].

## Supplementary Materials

The following supporting information can be downloaded at: https://www.mdpi.com/article/10.3390/ijms241411457/s1.
