# Supplementary material for: A Proteomic Survey of the Cystic Fibrosis Transmembrane Conductance Regulator Surfaceome
Source: Int J Mol Sci. 2023 Jul 14;24(14):11457. doi: 10.3390/ijms241411457 (PMC10380767; doi:10.3390/ijms241411457)
Supplement: Supplementary file 1 [file ijms-24-11457-s001.zip › figure s2.pdf]

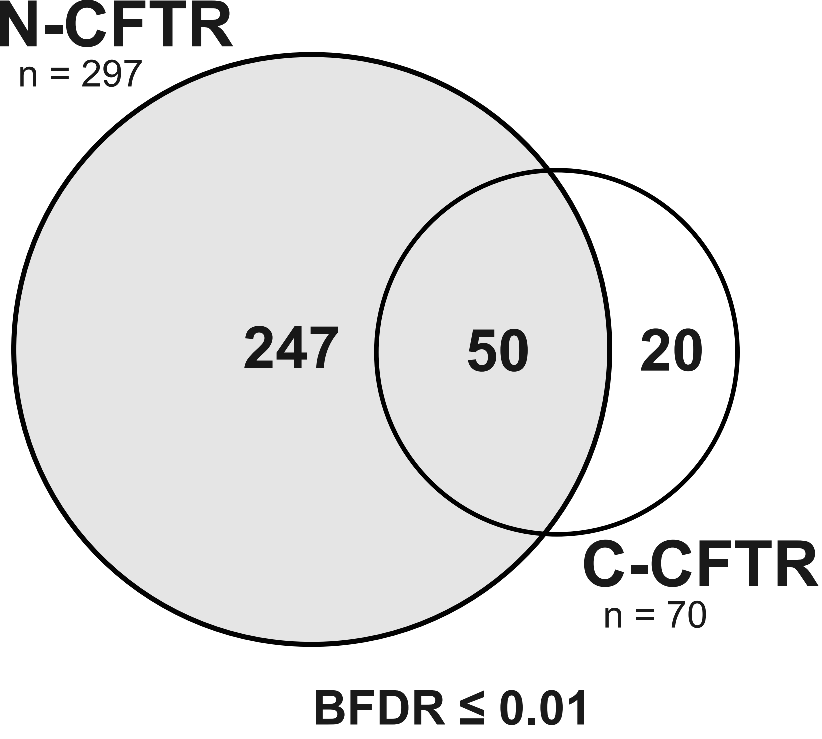

(a)

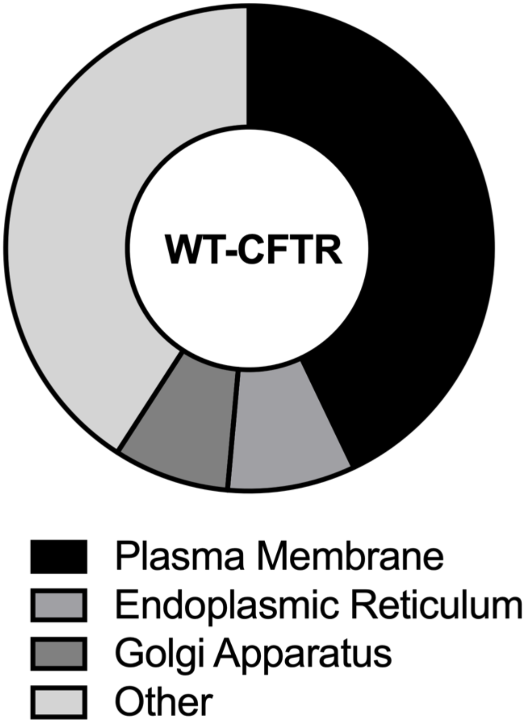

(b)

Preys in GO Category: Plasma Membrane

N-CFTR					C-CFTR
ATP2B1	**GOPC**	ITGA2	**SLC9A3R2**	**STX6**	P4HB
CALR	GPRC5A	ITGB1	SNX27	SCRIB	SCRIB
CAV1	IRS4	**SLC9A3R1**	SSR1		

(c)
