# Supplementary figures and images for: A Proteomic Survey of the Cystic Fibrosis Transmembrane Conductance Regulator Surfaceome
Source: Int J Mol Sci. 2023 Jul 14;24(14):11457. doi: 10.3390/ijms241411457 (PMC10380767; doi:10.3390/ijms241411457)

Pankow et al.

Reilly et al.

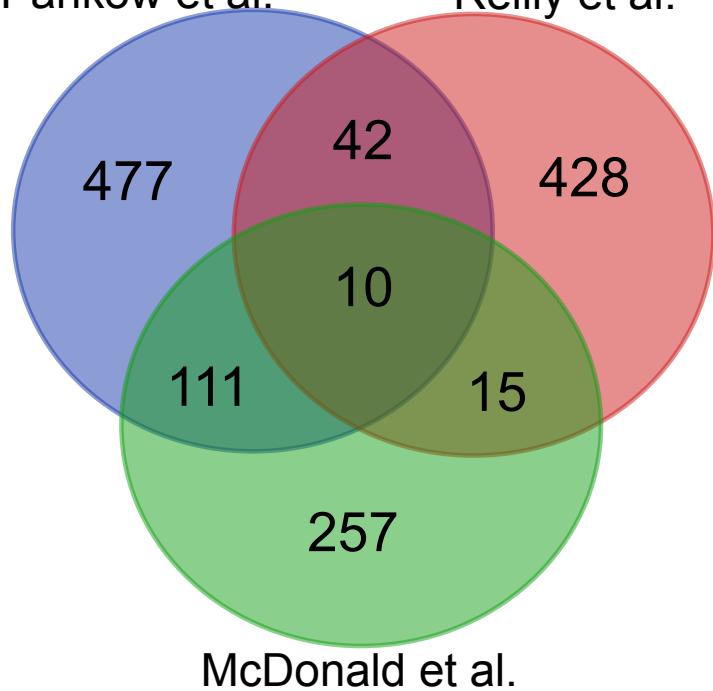

(a)

BioID

TurboID

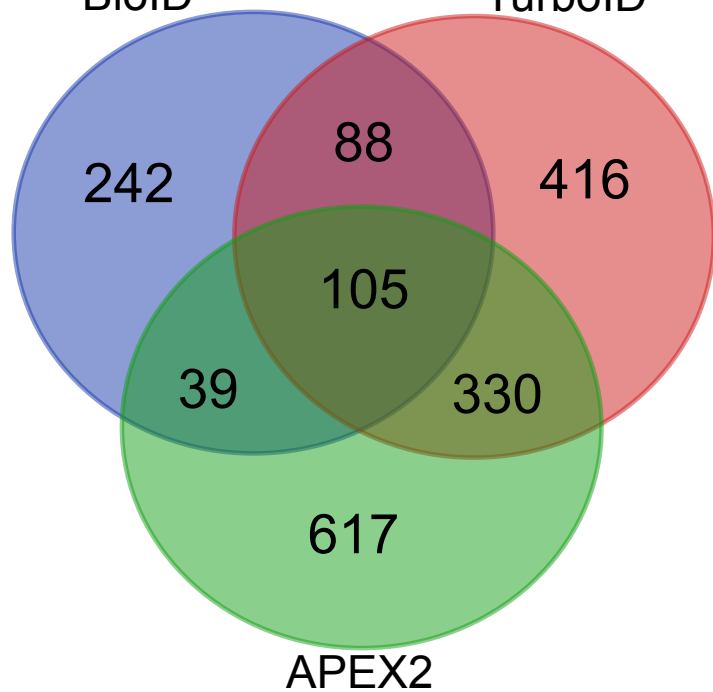

(b)

Supplement: Supplementary file 1 [file ijms-24-11457-s001.zip › figure s1.pdf]
